# Comparison of the clinical manifestations between different age groups of patients with overseas imported COVID-19

**DOI:** 10.1371/journal.pone.0243347

**Published:** 2020-12-04

**Authors:** Yujuan Han, Zujin Luo, Wenliang Zhai, Yue Zheng, Huan Liu, Yanran Wang, Endong Wu, Fang Xiong, Yingmin Ma

**Affiliations:** 1 Department of Internal Medicine, Beijing Xiaotangshan Hospital, Beijing, China; 2 Department of Respiratory and Critical Care Medicine, Beijing Engineering Research Center of Respiratory and Critical Care Medicine, Beijing Institute of Respiratory Medicine, Beijing Chao-Yang Hospital, Capital Medical University, Beijing, China; 3 Emergence Department, Xuanwu Hospital, Capital Medical University, Beijing, China; 4 Department of Surgical Intensive Care Unit, Beijing Chao-Yang Hospital, Beijing, China; 5 Department of Health Education, Beijing Xiaotangshan Hospital, Beijing, China; 6 Department of Nephrology, Children’s Hospital, Capital Institute of Pediatrics, Beijing, China; 7 Critical Care Medicine Department, Beijing Chest Hospital, Capital Medical University, Beijing, China; 8 Oncology Department, Beijing Youan Hospital, Capital Medical University, Beijing, China; University of Mississippi Medical Center, UNITED STATES

## Abstract

The current study investigated the clinical manifestations and outcomes of different age groups of patients with overseas imported COVID-19. In total, 53 COVID-19 patients admitted to the designated Beijing Xiaotangshan Hospital between March 16 and April 15 of 2020 were included. Based on the percentage of disease aggravation during hospital stay according to CT, the patients were divided into two groups: ≤40 years (group A; n = 41) and >40 years (group B; n = 12). The demographic data, epidemiological history, disease courses, potential complications, clinical symptoms, lab indices, chest CT outcomes, treatment protocols and turnovers of the two groups were compared. According to clinical typing, compared with group A, group B had a significantly greater proportion of the common type of COVID-19 (P<0.05) and greater comorbidity of type 2 diabetes (P<0.001). The two groups presented significantly different lab indices. Group B showed significantly more frequent CT abnormalities, with greater proportions of multiple lesions and bilateral lung involvement (P<0.05). During hospitalization, group B had a greater proportion of disease aggravation according to CT (P<0.01). Compared with group A, group B received a significantly greater proportion of antiviral therapy and presented a significantly greater occurrence of adverse drug reactions (P<0.05). The two groups did not significantly differ in time from admission to clinical symptom improvement or from disease onset to negative outcomes according to nucleic acid testing, the appearance of IgG or the appearance of IgM. They also did not significantly differ in length of stay. Older imported COVID-19 patients, particularly those with type 2 diabetes, showed a broader pulmonary extent and faster development of the disease, more severe pathogenetic conditions and a greater risk of developing a critically severe type. Increased attention should be given to this population in clinical practice.

## Introduction

Coronavirus disease 2019 (COVID-19) refers to an acute respiratory infectious disease caused by severe acute respiratory syndrome coronavirus 2 (SARS-CoV-2) [1]. Today, COVID-19 continues to rapidly spread across the world [2–4], posing a great threat to the life and health of human beings. This condition has attracted great attention from scholars and researchers.

To date, numerous studies have been conducted and released from the perspectives of different facets of the disease, such as demographics, clinical manifestations and treatment measures [5–8]. Although populations of all ages are vulnerable to COVID-19, an older age and complications caused by other underlying diseases may increase the risk associated with this condition [9] and affect the prognosis. Currently, studies on overseas imported COVID-19 remain rare, and whether age affects the demographic characteristics, clinical manifestations, treatment methods and turnover of patients with imported COVID-19 has not been determined.

On the basis of the aforementioned information, this study compared the demographic characteristics, clinical manifestations, treatment methods and turnovers of patients with imported COVID-19 among different age groups. The results of this study may provide a more in-depth understanding of COVID-19 and therefore may have particular clinical significance.

## Patients and methods

### Study objects

Beijing Xiaotangshan Hospital serves as an institute designated for the treatment of patients with overseas imported COVID-19 in Beijing. In this study, 53 patients who were confirmed to have imported COVID-19 at this hospital between March 16 and April 15, 2020 were consecutively included. Among these patients, 21 were males and 32 were females. Their ages ranged from 1 year to 74 years, with a median of 23 years (S1 File). The diagnostic criteria for COVID-19 were in accordance with those described in the Diagnosis and Treatment of Novel Coronavirus Infection Pneumonia 2020 (seventh edition) enacted by the National Health Commission of China [10]. All patients were confirmed by positive results from novel coronavirus nucleic acid testing on respiratory samples.

The procedures of this study were approved by the Ethics Committee of Beijing Xiaotangshan Hospital (approval No. 2020-LSD-05). Considering the retrospective nature of this study, written informed consent was waived by the committee.

### Methods

**Fig 1:**
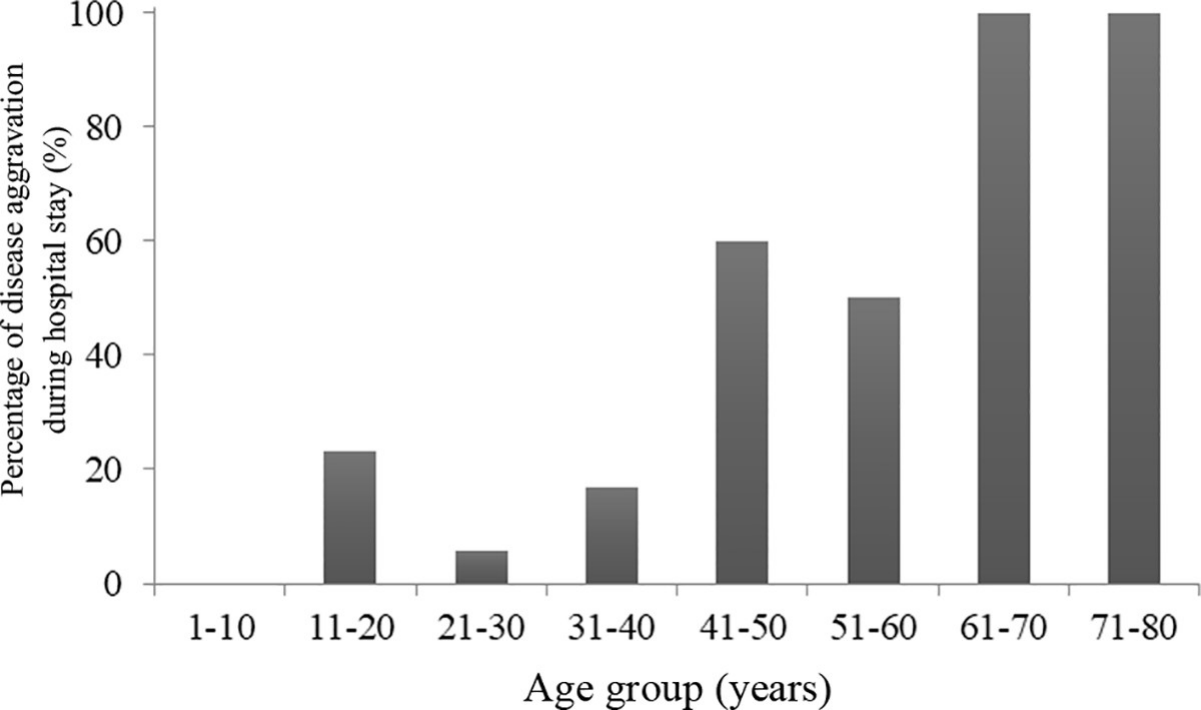
The percentage of COVID-19 aggravation along an age gradient.

Chest CT occupies an important position in COVID-19 screening; it also exhibits unique advantage in evaluating the severity of the disease [11, 12]. To investigate the influence of age on the clinical manifestations of overseas imported COVID-19, the percentages of disease aggravation of different age groups during hospital stay were compared according to CT observations. Specifically, we investigated this index by age subgroup with 10-year intervals. The two age groups on the ends of the spectrum were 1–10 years and 71–80 years, and the percentages of disease aggravation were noticeably lower in the age subgroups of 1–40 years, compared with the subgroups of 41–80 years (Fig 1). Considering a small number of the cases in this study, the included patients were divided into a ≤40-year-old group (group A; n = 41) and a >40-year-old group (group B; n = 12). The demographic data, epidemiological history, disease courses, potential complications, clinical symptoms, laboratory outcomes, chest CT outcomes, treatment protocols and turnovers of the two groups were collected. In addition, the outcomes of the first blood examination after admission were collected (S1 File), which included (1) routine blood tests, including white blood cell counts, neutrophilic granulocyte counts, lymphocyte counts, neutrophilic granulocyte percentages, hemoglobin percentages and platelet percentage; (2) biochemical indices of liver and renal functions, including alanine aminotransferase (ALT), aspartate aminotransferase (AST), total bilirubin, albumin, serum creatinine, lactate dehydrogenase, creatine kinase isoenzyme, troponin, blood glucose, triglycerides and prothrombin time; (3) arterial blood gas analysis, including the pH value, arterial oxygen partial pressure and carbon dioxide partial pressure; and (4) others, including C-reactive protein (CRP), procalcitonin and the erythrocyte sedimentation rate (ESR). The imaging data included CT images obtained at the time of admission or from the screening ward.

### Statistical analysis

Statistical analysis was performed using SPSS (version 25.0, SPSS, Chicago, IL, USA). Continuous variables with a normal distribution are presented as the means ± standard deviations (SDs), and those abnormally distributed are presented as the medians (25th-75th percentiles), unless otherwise specified. Qualitative or categorical variables are presented as absolute values with percentages. Normality was assessed using the Kolmogorov–Smirnov test. For continuous variables, Student’s *t* test or the Mann-Whitney U test was used for comparisons between groups. Qualitative or categorical variables were compared using the chi-squared test with (1 < theoretical frequency ≤5) or without continuity correction (theoretical frequency > 5) or Fisher’s exact test (theoretical frequency ≤ 1). All tests were two-sided, and differences with P<0.05 were considered statistically significant. However, considering the data driven definition of the groups and the small sample size of this exploratory study, the P-values in this study are provided for descriptive purposes only.

## Results

### General data

The general data of the two groups are summarized in Table 1. Among the 53 included patients, 26 were from the UK, while the rest were from other countries, with a total of 21 males and 32 females. Group A and group B did not significantly differ in country of origin or the sex ratio. According to the disease severity at the time of admission, 21 cases were mild, and 32 were moderate. Additionally, the proportion of the moderate type in group B was significantly greater than that in group A (P<0.05). All patients were from epidemic areas, and 32 patients had a history of contact with confirmed COVID-19 patients. Clustering of the disease occurred in three families (including 5 pediatric patients). Additionally, 3 patients were complicated with hypertension, 2 were complicated with type 2 diabetes (T2DM), and 1 was complicated with coronary artery disease. Compared with group A, group B had a significantly greater T2DM incidence (P<0.001). Regarding the APACHE II, NEWS2 and SIRS tests at the time of admission, group B had significantly greater scores than did group A.

**Table 1:** General data of the two groups.

Item	Group A (n = 41)	Group B (n = 12)	Z/χ2	P
Characteristic				
Country (UK/Others)	21 (51%)/20 (49%)	5 (42%)/7 (58%)	0.339	0.560
Sex (M/F)	16 (39%)/25 (61%)	5 (42%)/7 (58%)	0.027	0.869
Clinical classification (MI/MO)	20 (49%)/21 (51%)	1 (8%)/11 (92%)	6.348	0.012
Epidemiologic				
Exposure to confirmed or suspected patients	24 (59%)	7 (58%)	0.0002	0.990
Agglomerative disease	17 (41%)	6 (50%)	0.275	0.600
Comorbidities				
Hypertension	1 (2%)	2 (17%)	3.519	0.061
Diabetes	0 (0%)	4 (33%)	14.782	<0.001
Coronary heart disease	0 (0%)	1 (8%)	3.482	0.062
Current smoker	5 (12%)	1 (8%)	0.138	0.710
Onset of symptoms to hospital admission, days	3 (1–7)	6 (2–8)	-0.827	0.408
Severity-status scores				
qSOFA	0 (0–1)	0 (0–1)	-1.03	0.303
SOFA	0 (0–1)	0 (0–2)	-1.379	0.168
APACHE II	1 (0–2)	3 (2–3)	-3.023	0.003
CURB-65	0 (0–0)	0 (0–0)	-1.03	0.303
NEWS2	1.5 (0–2.25)	3 (2–4)	-2.534	0.011
SIRS	1 (0–2)	2 (2–3)	-3.428	0.001

The P-values are provided for descriptive purposes only in this study because of the small sample size. UK, United Kingdom; M, male; F, female; MI, mild; MO, moderate.

### Clinical symptoms

Among the 53 patients, fever was the most frequent symptom (22 cases, 42%), followed by dry cough (17 cases, 32%), sore throat (15 cases, 28%), headache (14, 26%) and throat dryness (12, 23%). Rare symptoms included chills, expectoration, myalgia or arthralgia, hyposmia, dizziness, chest pain, rhinorrhea, nausea, diarrhea, and so on. In group A, the most frequent symptoms included fever (17 cases, 41%), sore throat (14, 34%) and dry cough (13, 32%). In group B, the most frequent symptoms included fever (5, 42%), dry cough (4, 33%) and dry pharynx (4, 33%). The two groups did not differ significantly in frequency of symptoms (P>0.05). The average maximum body temperature and arterial pressure of group B on the admission day were noticeably greater than those of group A (P<0.05) (Table 2).

**Table 2:** Clinical symptoms and body signs of the two groups.

Clinical signs and symptoms	Group A (n = 41)	Group B (n = 12)	Z/χ2	P
Fever	17 (41%)	5 (42%)	0.0002	0.990
Chills	3 (7%)	3 (25%)	2.891	0.089
Cough	13 (32%)	4 (33%)	0.011	0.915
Sputum	9 (22%)	2 (17%)	0.158	0.691
Chest pain	6 (15%)	0 (0%)	1.98	0.159
Dyspnea	0 (0%)	0 (0%)		
Nasal congestion	6 (15%)	2 (17%)	0.03	0.863
Rhinorrhea	8 (20%)	2 (17%)	0.049	0.825
Throat dryness	8 (20%)	4 (33%)	1.102	0.314
Sore throat	14 (34%)	1 (8%)	3.048	0.081
Headache	11 (27%)	3 (25%)	0.016	0.899
Dizziness	5 (12%)	0 (0%)	1.616	0.204
Fatigue	4 (10%)	2 (17%)	0.442	0.506
Myalgia or arthralgia	6 (15%)	2 (17%)	0.03	0.863
Nausea or vomiting	2 (5%)	1 (8%)	0.208	0.649
Anorexia	3 (7%)	3 (25%)	2.891	0.089
Diarrhea	6 (15%)	1 (8%)	0.321	0.571
Hyposmia	6 (15%)	3 (25%)	0.708	0.400
Hypogeusia	6 (15%)	1 (8%)	0.321	0.571
T_max_ (°C)	36.9 (36.7–37.2)	37.4 (36.9–38.6)	-2.434	0.015
Respiratory rate (/min)	20 (20–22)	21 (20–22)	-1.839	0.066
SPO2_min_ (%)	97 (96–98)	97 (96–98)	-1.19	0.234
HR (/min)	90 (80–104.75)	100 (91–106)	-1.602	0.109
MAP (mmHg)	92.15 (86.75–98)	99 (90–107)	-2.063	0.039

The P-values are provided for descriptive purposes only in this study because of the small sample size. T_max_, maximum body temperature on the day of admission; HR, heart rate; MAP, mean arterial pressure; SPO2_min_, minimum pulse oxygen saturation.

### Laboratory indices

The two groups did not significantly differ in peripheral-blood white blood cell count, neutrophilic granulocyte count, lymphocyte count, percentage of neutrophilic granulocytes, hemoglobin, total bilirubin, serum creatinine, troponin, creatine kinase isoenzyme, activated prothrombin time, D-dimer, procalcitonin, or carbon dioxide partial pressure. The lymphocyte percentage, platelets, albumin, lactate dehydrogenase, triglyceride, prothrombin time, INR, and arterial oxygen partial pressure in group B were significantly lower than those in group A, whereas the neutrophil-to-lymphocyte ratio, ALT, AST, blood glucose, PTA, fibrinogen, CRP, ESR, and pH value significantly increased (in particular the blood glucose and CRP) in group A (Table 3).

**Table 3:** Laboratory examination outcomes of the two groups.

Laboratory findings	Group A (n = 41)	Group B (n = 12)	Z/χ2	P
WBC (×10^9^/L)	5.10 (3.90–6.15)	4.21 (3.90–6.80)	-0.241	0.809
NEU (×10^9^/L)	2.39 (1.78–3.63)	3.04 (1.76–4.59)	-0.636	0.525
NEU (%)	51.20 (43.48–57.53)	57.70 (45.80–62.1)	-1.327	0.185
LYM (×10^9^/L)	2.04 (1.48–2.52)	1.75 (1.22–1.97)	-1.700	0.089
LYM (%)	37.65 (33.38–46.98)	31.30 (27.9–35.20)	-2.051	0.040
N/L	1.41 (0.87–1.69)	1.92 (1.44–2.76)	-2.215	0.027
HG (g/L)	138.50 (127.75–147.25)	136 (122–147)	-0.274	0.784
PLT (×10^9^/L)	230 (185.50–264.50)	190 (108–197)	-2.281	0.023
ALB (g/L)	43.90 (43.08–45.60)	41.80 (40.70–42.90)	-2.314	0.021
ALT (U/L)	15.50 (11.70–21.33)	18.80 (16.60–32.20)	-2.204	0.027
AST (U/L)	18.60 (15.95–21.33)	24.70 (18.00–30.40)	-2.194	0.028
TB (mmol/L)	9.67 (6.68–12.90)	8.20 (6.39–10.18)	-0.724	0.469
CR (μmol/L)	62.85 (56.43–76.98)	69.10 (59.40–94.40)	-1.228	0.219
LDH (U/L)	163.30 (142.13–202.93)	185.60 (175.60–219.40)	-2.237	0.025
TNI (pg/Ml)	0.01 (0.01–0.01)	0.01 (0.01–0.01)	-1.954	0.051
CK-MB (U/L)	9.50 (7.98–12.13)	10.80 (8.50–17.7)	-1.141	0.254
GLU (mmol/L)	4.89 (4.59–5.08)	5.18 (4.95–6.09)	-2.698	0.007
TG (mmol/L)	0.80 (0.61–0.98)	0.98 (0.83–1.21)	-2.128	0.033
PT (s)	13.70 (13.40–14.20)	13.20 (13.10–13.60)	-2.210	0.027
PTA (%)	93 (85–97)	100 (95–103)	-2.318	0.020
INR	1.04 (1.02–1.10)	1.00 (0.99–1.03)	-2.268	0.023
APTT (s)	38.95 (36.23–42.38)	43.20 (38.40–46.10)	-1.393	0.164
D-dimer (mg/L)	235 (220–342.5)	290 (220–420)	-0.71	0.478
FIB (g/L)	2.85 (2.57–3.53)	3.58 (3.07–4.22)	-2.500	0.012
PCT (ng/Ml)	0.03 (0.03–0.45)	0.05 (0.03–0.06)	-1.659	0.097
CRP (mg/L)	0.84 (0.17–3.73)	10.07 (1.22–21.58)	-2.983	0.003
ESR (mm/h)	8.50 (5.00–17.00)	19.00 (11.00–25.00)	-2.319	0.020
PH	7.36 (7.34–7.38)	7.39 (7.37–7.42)	-2.414	0.016
PaO_2_ (mmHg)	98.80 (88.80–104.30)	84.40 (75.30–93.10)	-2.564	0.010
PaCO_2_ (mmHg)	41.90 (39.00–45.30)	40.10 (38.20–43.50)	-1.218	0.223

The P-values are provided for descriptive purposes only in this study because of the small sample size. WBC, white blood cell count; NEU, neutrophil count; LYM, lymphocyte count; HG, hemoglobin; PLT, platelet count; ALB, albumin; ALT, alanine aminotransferase; AST, aspartate aminotransferase; TB, total bilirubin; CR, creatinine; LDH, lactate dehydrogenase; TNI, cardiac troponin I; CK-MB, creatine kinase muscle-brain isoform; PT, prothrombin time; PCT, procalcitonin; CRP, C-reactive protein; APTT, activated partial thromboplastin time; FIB, fibrinogen; ESR, erythrocyte sedimentation rate.

### Imaging outcomes

No abnormalities were observed among the 22 patients with mild COVID-19 according to chest CT. Compared with group A, group B showed a significantly greater incidence of abnormalities (P<0.01). The abnormalities were primarily manifested by ground-glass opacification (GGO) and “paving stone”-like changes with consolidation changes. The multilesion and bilateral involvement percentages of group B were significantly greater than those of group A (P<0.05). Given that the age split (40 years) between the groups was driven by CT-based observations of clinical aggravation during hospital stay, group B had a significantly higher percentage of disease aggravation during the hospital stay and a significantly higher CT score than group A (both P<0.01) (Table 4).

**Table 4:** Imaging outcomes of the two groups.

Radiologic findings	Group A (n = 41)	Group B (n = 12)	Z/χ2	P
Chest CT abnormality	20 (49%)	11 (92%)	10.460	0.008
Ground-glass opacities	17 (41%)	9 (75%)	4.178	0.041
Consolidation	9 (22%)	5 (42%)	1.856	0.173
Paving stone changes	4 (10%)	6 (50%)	9.821	0.002
Mixed lesions	4 (10%)	3 (25%)	1.882	0.170
Lesion distribution				
Local	13 (32%)	3 (25%)	0.198	0.656
Multifocal	7 (17%)	7 (58%)	8.130	0.004
Unilateral	16 (39%)	5 (42%)	0.027	0.869
Bilateral	4 (10%)	5 (42%)	6.705	0.009
CT exacerbations occurred during hospitalization	5 (12%)	8 (67%)	14.879	<0.001
Total CT score	0.50 (0.00–1.25)	2.00 (1.00–5.00)	-2.887	0.004

The P-values are provided for descriptive purposes only in this study because of the small sample size.

### Treatment and turnover

Detailed information on treatments and turnovers is summarized in Table 5.

**Table 5:** Treatment and turnover of the two groups.

Item	Group A (n = 41)	Group B (n = 12)	Z/χ2	P
Antiviral treatment	28 (68%)	12 (100%)	5.041	0.024
Antibiotics	7 (17%)	5 (42%)	3.206	0.073
Traditional Chinese medicine	33 (80%)	12 (100%)	2.758	0.097
Corticosteroids	0 (0%)	0 (0%)		
Immunomodulators	4 (10%)	2 (17%)	0.442	0.506
Adverse drug reactions	7 (17%)	6 (50%)	5.437	0.020
Nasal cannula oxygen therapy	0 (0%)	4 (33%)	14.782	0.0001
High-flow nasal cannula oxygen therapy	0 (0%)	0 (0%)		
Severe COVID-19	4 (10%)	6 (50%)	9.821	0.002
Time to clinical improvement	14 (6–20)	13 (8.25–23)	-0.468	0.640
Time from illness onset to viral shedding	11 (7–15.25)	14 (11–28)	-1.356	0.175
Time from illness onset to positive serum immunoglobulin G	15 (11–20)	13 (10.25–17.5)	-0.695	0.487
Time from illness onset to positive serum immunoglobulin M	20 (16–27)	18.5 (11–23.75)	-0.737	0.461
Transfer for advanced treatment	2	4	7.487	0.006
Hospital stay	14 (11–19)	17 (11.75–28)	-0.852	0.394
Live discharge	39	8	7.487	0.006

The P-values are provided for descriptive purposes only in this study because of the small sample size.

Among the included patients, a total of 40 received antiviral treatment, 12 received antibiotic treatment, 45 received traditional Chinese medicine treatment, and 6 received immune preparations. No patients were subjected to hormone treatment. Compared with group A, group B significantly differed in the percentage of patients receiving antiviral treatment (P<0.05). A total of 13 patients presented with adverse drug effects, with the incidence in group B significantly greater than that in group A (P<0.05). Four patients were subjected to nasal oxygen inhalation treatment due to disease aggravation, and all of them were in group B. Ten patients presented with aggravated disease severity after admission, with the incidence in group B significantly greater than that in group A ((6, 50%) vs. (4, 10%); P<0.05). The two groups did not significantly differ in the time span from admission to clinical symptom improvement, from disease onset to negative outcomes according to nucleic acid testing, from disease onset to the appearance of IgG or from disease onset to the appearance of IgM. They also did not significantly differ in length of stay. Six patients had to be transferred to another hospital due to disease aggravation, and a significant difference was observed between group B (4 patients; 33%) and group A (2 patients; 5%) (P<0.01). No patients presented with complications during the hospital stay. No deaths occurred in either group.

## Discussion

In this study, we reported the clinical characteristics of 53 patients with overseas imported COVID-19 who received treatment at the designated Beijing Xiaotangshan Hospital. Their demographic characteristics, clinical manifestations, imaging examination results, laboratory testing results, treatment methods, and turnovers were analyzed. For the first time, this study investigated whether different age groups of imported COVID-19 differed in terms of these analyzed indices. Comparisons between the two groups revealed that group B presented with more severe pathogenetic conditions. Although the two groups did not significantly differ in clinical symptoms, they did show significant differences in terms of laboratory examination outcomes, imaging outcomes, and disease aggravation during hospitalization.

In both groups, there were more females than males. However, there was no significant difference in the sex ratio between the two groups. Compared with group A, Group B showed a significantly greater percentage of moderate cases. The average body temperature on the day of admission of group B was greater than that of group A. These findings suggested that, compared with group A, group B had more severe pathogenetic conditions and that age might be an independent risk factor for the disease. These suggestions were also supported by the finding that the APACHE II, NEWS2 and SIRS scores of group B were greater than those of group A [13, 14]. In this study, all patients were from epidemic areas, and clustering of the disease occurred in three families, including 5 pediatric patients. Pediatric patients have previously been reported in family clustering of COVID-19 [4], and they account for nearly 1% of all reported COVID-19 patients in China [15]. These findings suggest that populations of all ages are vulnerable to COVID-19. The two groups did not significantly differ in complications associated with hypertension and coronary arterial disease, but they significantly differed in the incidence of T2DM complications. Therefore, in clinical practice, clinicians should be alerted to the possibility of COVID-19 aggravation in elderly patients complicated with T2DM. In addition, the occurrences of vomiting and diarrhea in this study were 6% and 13%, respectively, which are lower than those reported in the literature [16, 17]. The reason for this difference might be that patients with severe and critically severe COVID-19 were not included in this study. Furthermore, no patients presented with dyspnea in this study.

Compared with group A, group B showed a noticeably decreased lymphocyte percentage, which indicated that the virus may act on lymphocytes [18]. The two groups also significantly differed in the increase of the CRP level. CRP serves as an important index of systemic inflammatory reactions. An increase in the CRP level, as well as the extent of the increase, may be associated with the severity of COVID-19 [19]. In this study, 22% of the patients presented with an increased CRP level at the time of admission, which suggested that an increase in the CRP level may occur at an early stage of COVID-19. Comparisons between group A and group B showed that patients with a decreased lymphocyte percentage, increased CRP, liver function abnormalities, increased lactate dehydrogenase and decreased arterial partial pressure of oxygen are at greater risk of disease progression. These findings may contribute to the early identification of patients whose diseases may convert to severe types. One of the important pathophysiological characteristics of COVID-19 is functional disorders of the coagulation-fibrinolysis system. These disorders may be associated with the inflammatory storms induced by viral infection, and a prolonged prothrombin time (PT) and increased D-D have been shown to occur in patients with severe COVID-19, as well as in patients who have died from the disease [2, 20]. However, in this study, the PT of group B was significantly shorter than that in group A. Although the D-D value of group B was greater than that of group A, no significant difference was observed between the two groups. Our findings are inconsistent with those reported in the literature [2, 13]. Presumably, the reason for these inconsistencies is that this study and those in the literature included different groups of patients; that is, only patients with mild and moderate COVID-19 were included in this study. According to imaging examination, COVID-19 patients exhibit typical characteristics. At an early stage, the CT images are characterized by multiplaque GGOs distributed at the lobular core below the pleura, whose inside texture may exhibit reticular changes [20, 21]. At the advanced stage, the images are characterized by an increased number of lesions with an expanded extent and the coexistence of GGO and consolidation or strip opacification. At the severe stage, consolidation is the main manifestation on CT images. The results of this study showed that older patients were at a greater risk of aggravation to severe COVID-19, with a larger extent of lesions and more severe pathogenetic conditions. Patients with critically severe COVID-19 are mostly elderly patients, and elderly patients complicated with underlying diseases, such as hypertension and diabetes, are at a greater risk of death, which indicates that elderly patients are the greatest-risk population of COVID-19 [22]. Therefore, for elderly patients, frequent CT examination at the early stages of treatment is helpful to best identify those who are likely to develop severe COVID-19.

To date, no COVID-19-specific medicine has been developed, and symptomatic supportive treatment remains the main method for COVID-19 patients [23]. Comparisons between group A and group B suggested that, compared with younger patients, older patients tend to have more severe pathogenetic conditions; older patients are at a greater risk of disease aggravation to severe and critically severe types and are more likely to present with adverse drug effects, which possibly further aggravate the condition. Therefore, for this population, medication should be given cautiously. In addition, 4 of the discharged patients were rehospitalized due to positive relapse during separation. The positivity rate of sputum nucleic acid tests was greater than that of throat swab sample tests in these patients. This finding suggested that there might be virus residue in the lower respiratory tract. To reduce the possibility of nucleic acid positivity relapse, both sputum and throat swabs are suggested for detection (however, this treatment may prolong the time needed for nucleic acid testing outcomes to appear negative). The two groups did not significantly differ in terms of the time from admission to clinical symptom improvement, from disease onset to negative outcomes according to nucleic acid testing, from disease onset to the appearance of IgG or from disease onset to the appearance of IgM.

This study has some important limitations. First, this exploratory study was single-centered and retrospective, with a data-driven split into groups at age 40. Therefore, future studies with a priori hypotheses regarding the role of age in oversease imported COVID-19 in this population need to be conducted to confirm our observations. Second, the small sample size of this study was small. In the future, studies with a larger sample size of imported COVID-19 need to be conducted. Third, all included patients were subject to overseas imported COVID-19, and therefore, the applicability of the results of this study to local patients needs to be verified. Fourth, for the patients transferred to other hospitals because of disease aggravation, there was a lack of prognostic assessments and follow-up results, and therefore, more data need to be accumulated. Fifth, all patients were statistically analyzed as independent individuals in this study. However, there were patients from the same family (family clustering), which might increase the risk of first-class errors in this study. That is, the acutual P values might be larger than the estimated P values in this study. Last, this study did not involve patients with severe and critically severe COVID-19. In the future, patients with severe and critically severe imported COVID-19 will be included, whereby comprehensive comparative studies can be carried out to investigate the possible differences between imported cases and local cases.

In conclusion, compared with their younger counterparts, older patients with imported COVID-19, particularly those complicated with T2DM, tend to present with a broader extent and faster advancement of pulmonary lesions. They have more severe pathogenetic conditions, are at a greater risk of aggravation to severe and critically severe types, and have a poorer prognosis than do younger patients. For these patients, intense monitoring of pathogenetic conditions should be performed to avoid treatment delays.

## Supporting information

S1 File General data, the outcomes of CT examination, nucleic acid testing and antibody detection, and disease turnover. (XLSB)
